# Inhibition of SARS-CoV-2 by Highly Potent Broad-Spectrum Anti-Coronaviral Tylophorine-Based Derivatives

**DOI:** 10.3389/fphar.2020.606097

**Published:** 2020-12-14

**Authors:** Cheng-Wei Yang, Yue-Zhi Lee, Hsing-Yu Hsu, Jia-Tsrong Jan, Yi-Ling Lin, Sui-Yuan Chang, Tzu-Ting Peng, Ruey-Bing Yang, Jian-Jong Liang, Chun-Che Liao, Tai-Ling Chao, Yu-Hau Pang, Han-Chieh Kao, Wen-Zheng Huang, Jiunn-Horng Lin, Chun-Ping Chang, Guang-Hao Niu, Szu-Huei Wu, Huey-Kang Sytwu, Chiung-Tong Chen, Shiow-Ju Lee

**Affiliations:** ^1^ Institute of Biotechnology and Pharmaceutical Research, National Health Research Institutes, Miaoli, Taiwan; ^2^ Genomic Research Center, Academia Sinica, Taipei, Taiwan; ^3^ Institute of Biomedical Sciences, Academia Sinica, Taipei, Taiwan; ^4^ Institute of Clinical Laboratory Sciences and Medical Biotechnology, College of Medicine, National Taiwan University, Taipei, Taiwan; ^5^ Animal Technology Laboratories, Agricultural Technology Research Institute, Hsinchu, Taiwan; ^6^ National Institute of Infectious Diseases and Vaccinology, National Health Research Institutes, Miaoli, Taiwan

**Keywords:** HCoV-OC43, HCoV-229E, FIPV, tylophorine, SARS-CoV-2, ouabain, COVID-19, coronavirus

## Abstract

Tylophorine-based compounds and natural cardiotonic steroids (cardenolides and bufadienolides) are two classes of transmissible gastroenteritis coronavirus inhibitors, targeting viral RNA and host cell factors, respectively. We tested both types of compounds against two types of coronaviruses, to compare and contrast their antiviral properties, and with view to their further therapeutic development. Examples of both types of compounds potently inhibited the replication of both feline infectious peritonitis virus and human coronavirus OC43 with EC_50_ values of up to 8 and 16 nM, respectively. Strikingly, the tylophorine-based compounds tested inhibited viral yields of HCoV-OC43 to a much greater extent (7–8 log magnitudes of p.f.u./ml) than the cardiotonic steroids (about 2–3 log magnitudes of p.f.u./ml), as determined by end point assays. Based on these results, three tylophorine-based compounds were further examined for their anti-viral activities on two other human coronaviruses, HCoV-229E and SARS-CoV-2. These three tylophorine-based compounds inhibited HCoV-229E with EC_50_ values of up to 6.5 nM, inhibited viral yields of HCoV-229E by 6–7 log magnitudes of p.f.u./ml, and were also found to inhibit SARS-CoV-2 with EC_50_ values of up to 2.5–14 nM. In conclusion, tylophorine-based compounds are potent, broad-spectrum inhibitors of coronaviruses including SARS-CoV-2, and could be used for the treatment of COVID-19.

## Introduction

COVID-19, the disease caused by infection with SARS-CoV-2, has now spread to more than 187 countries, areas or territories; infected 26,011,421 people; and caused 863,966 deaths with a global mortality rate of 3.32% (as of September 03, 2020, https://www.cdc.gov.tw/) since the first reports of patient cases emerged from WuHan, China in December 2019 (Ali and Alharbi, 2020; Velavan and Meyer, 2020). COVID-19 has been the subject of frenzied research activity, and a handful of potential treatments have now been identified, including novel drugs such as remdesivir (Amirian and Levy, 2020), and repurposed older ones such as ciclesonide (Iwabuchi et al., 2020), chloroquine (Gao et al., 2020; Touret and de Lamballerie, 2020), and the combination of hydroxychloroquine and azithromycin (Gautret et al., 2020a; Gautret et al., 2020b). However, there remains an urgent unmet need for additional therapies, especially given case reports of COVID-19 patients who recovered from the disease only to test positive again—perhaps due to their discontinuation of antiviral drugs (Wu et al., 2020); or their viral load being below the threshold of detection and/or restricted to particular tissues, and therefore difficult to detect with regular diagnostic methods (Hoang et al., 2020; Kang et al., 2020; Long et al., 2020). Accordingly, drugs that directly target the SARS-CoV-2 virus and diminish the patient’s viral load alone or in combination with other drugs should be further pursued, in addition to those designed to mitigate the symptoms of COVID-19.

In previous work, we established the potent anti-coronaviral activity of tylophorine-based compounds against transmissible gastroenteritis virus (TGEV), severe acute respiration syndrome coronavirus (SARS-CoV), or murine hepatitis virus (MHV) (Yang et al., 2010; Lee et al., 2012; Yang et al., 2017a); and cardiotonic steroids (cardenolides and bufadienolides), against TGEV but not MHV (Yang et al., 2017a). The underlying mechanism of action for these two classes of antiviral compounds are different: tylophorine based compounds target the viral ribonucleoprotein complex (Yang et al., 2017b), whereas cardiac steroids interfere with host factors via either augmenting PI3K_PDK1 signaling or downregulating JAK1 (Yang et al., 2017a; Yang et al., 2018; Yang et al., 2020a).

Here, we tested these tylophorine-based compounds and cardiotonic steroids against a wider variety of coronaviruses (feline inflammatory peritonitis virus (FIPV) and the human coronaviruses HCoV-OC43, HCoV-229E, and SARS-CoV-2) and assessed their effect on viral yields/load by end point and TCID50 assays (Reed and Muench, 1938), which determine the infectious titer over a period of 4 (for HCoV-229E) or 6 (for HCoV-OC43) days. All of the compounds tested were found to be highly potent inhibitors of HCoV-OC43 (low EC_50_ values at low nM concentration ranges), as measured by IFA against viral nucleocapsid protein, but their safety indices and the extent to which they diminished viral yields were more variable. Finally, we demonstrated that the tylophorine-based compounds are highly potent inhibitors of SARS-CoV-2, and therefore may be of merit as treatments for COVID-19.

## Materials and Methods

### Chemicals and Antibodies for IFA Analyses

DMSO (≧99.5%), digoxin (D6003, ≧95%, HPLC), digitoxin (D5878, ≧92%, HPLC), digitoxigenin (D9404, 99%, TLC), ouabain (O3125, ≧95%, HPLC), oleandrin (O9640, ≧98%, HPLC), crystal violet (C0775, dye content ≥90%), and methylcellulose (#M0387) were purchased from Sigma-Aldrich (St. Louis, MO, United States); bufalin (15725, ≧ 98%, HPLC) from Cayman Chemical (Ann Arbor, MI, United States); digoxin-BSA (80-ID10, HPLC) from Fitzgerald Industries (Acton, MA, United States); rostafuroxin (T2621, ≧99%, HPLC) from Target Molecule Corp*.* (Boston, MA*,* United States); istaroxime hydrochloride (HY-15718A, ≧99%, HPLC) from MedChem Express (Monmouth Junction, NJ, United States); and Remdesivir (GS-5734) (S8932, 99.3%, HPLC) and GS-441524 (S6814, 99.3%, HPLC) were from Selleckchem (Houston, TX, United States). The antibody against nucleocapsid proteins of HCoV-OC43 (Mab9013) was purchased from Merck Millipore (Burlington, MA, United States), and fluorescein isothiocyanate (FITC)-conjugated anti-mouse immunoglobulin (#55499) from MP Biomedicals (Irvine, CA, United States). Anti-SARS-CoV-2 N protein antibodies were provided by Dr. An-Suei Yang of the Genomics Research Center, Academia Sinica. Goat anti-human IgG-Alexa Fluor 488 (A11013) and DAPI (D1306) were purchased from Invitrogen. 10% formaldehyde solution was purchased from Marcon™ Chemicals (#H121-08).

### Tylophorine Based Compounds

dbq33b (99.0%, HPLC), dbq33b4p7 (99.4%, HPLC), PI09 (95.5%, HPLC), PQ09 (95.5%, HPLC), dbq29a (98.0%, HPLC) and 031p13-3 (>95%, TLC) were synthesized as previously reported (Yang et al., 2010; Lee et al., 2012) with some modifications which will be published elsewhere. NMR data of all these compounds has been previously reported (Yang et al., 2010; Lee et al., 2012) except those of 031p13-3 and dbq33b4p7, which are disclosed below.

#### 031p13-3

Yellow crystal; ^1^H-NMR (300 MHz, CDCl_3_): 2.01–2.04 (1H, m), 2.20–2.26 (2H, m), 3.16 (1H,d, J = 15.0 Hz), 3.30–3.39 (1H, m), 3.45–3.54 (2H, m), 3.71–3.81 (2H, m), 3.93 (3H, s), 3.97 (3H, s), 3.98 (3H, s), 4.06 (3H, s), 4.09 (3H, s), 4.85 (1H, d, J = 15.3 Hz), 5.35 (1H, d, J = 15.0 Hz), 6.87 (1H, s), 7.08 (1H, s), 9.16 (1H, s). ESI-MS m/z 440 (M + H)^+^.

#### dbq33b4p7

White crystal; ^1^H-NMR (400 MHz, CDCl_3_): 1.01 (3H, t, J = 7.2 Hz), 1.41 (3H, d, J = 6.4 Hz), 2.52 (1H,qd, J = 6.4, 4.4 Hz), 2.78 (2H,qd, J = 7.2, 4.4 Hz), 3.12 (1H,d, J = 15.2 Hz), 3.28 (1H,d, J = 15.2 Hz), 4.66 (1H, s), 6.26 (1H, s), 7.21 (1H,dd, J = 9.2, 2.4 Hz), 7.06 (1H, s), 7.73 (1H,d, J = 2.4 Hz), 8.29 (1H,d, J = 2.4 Hz). ^13^C-NMR (150 MHz, CDCl_3_): 9.3, 15.8, 46.1, 51.3, 55.5, 55.7, 55.8, 57.3, 68.4, 102.8, 103.3, 104.4, 114.8, 123.9, 124.1, 124.6, 126.4, 128.7, 130.5, 148.6, 148.9, 157.6. LRMS (EI^+^) m/z (rel intensity) 381 (M^+^, 14%) and 310 (100%). HRMS calcd for C_23_H_27_NO_4_ (M^+^) 381.1940; found, 381.1930.

### Cells, Viruses, Immunofluorescent Assay, Cytopathic Effect, Plaque Assay and Cytotoxicity Assay

*Felis catus* whole fetus-4 (Fcwf-4) cells (ATCC^®^CRL-2787) were maintained in Dulbecco’s modified Eagle’s medium (DMEM, Hyclone Laboratories, Logan, UT, United States) containing 10% fetal bovine serum (FBS) with 1% penicillin/streptomycin at 37°C with 5% CO_2_. The serotype II FIPV Taiwan isolate NTU156 strain, a kind gift from National Taiwan University, was propagated and titrated in Fcwf-4 cells (Lin et al., 2009). The EC_50_ for anti-FIPV activity and CC_50_ for cell cytotoxicity were determined as previously described (Yang et al., 2020b).

Human colon adenocarcinoma cell line HCT-8 (ATCC^®^ CCL-244™) was obtained from American Type Culture Collection (ATCC) and passaged within 6 months of receipt. It was established as stock in the cell bank at an early passage, to ensure cell line-specific characteristics. HCoV-OC43 (ATCC^®^ VR1558™) was grown and propagated in HCT-8 cells cultured with DMEM and 2% FBS. For compound treatment studies, cells were seeded in 96-well plates and then cultured in DMEM medium containing 2% FBS. Cells were pretreated with compounds each in a series of five concentrations at five-fold dilution for 1 h prior to HCoV-OC43 infection at an MOI of 0.05. The supernatants at 72 h.p.i. from OC43 infected HCT-8 cells treated with the test compounds were subjected to the end-point assay and TCID50 determination at 6 d.p.i. to measure the viral-yield inhibition of each treatment. This procedure was performed to quantify how much infectious virus was present in a preparation. The resultant adherent cells (72 h.p.i.) were then fixed with 80% acetone and subjected to IFA analyses with an antibody against OC43 N protein and the EC_50_ values determined as described (Yang et al., 2020b). The viabilities of HCT-8 cells culture in media containing 10% FBS treated with compounds each in a series of eight concentrations at two-fold dilution for 72 h were determined using the CellTiter 96 AQueous Non-Radioactive Cell Proliferation Assay kit (MTS) (Promega, Madison, WI, United States); CC_50_ values were determined as previously described (Yang et al., 2020b). In addition, for visualization of cytopathic effects of HCoV-OC43 on HCT-8 cells infected at an MOI of 0.05, the cells were fixed by 80% acetone at 6 d.p.i. and stained with crystal violet.

Human lung fibroblasts cells, MRC-5 (ATCC^®^ CCL-171™) were cultured in Eagle’s Minimum Essential Medium (MEM) supplemented with 10% fetal bovine serum and penicillin/streptomycin at 37 °C with 5% CO_2_. HCoV-229E (ATCC^®^ VR-740™) were grown and propagated in MRC-5 cells cultured with MEM and 2% FBS. For the compound treatment studies, cells were then cultured in MEM medium containing 2% FBS. MRC-5 cells were seeded the day before compound treatment and HCoV-229E infection. The tested compounds were added to the wells 1 h prior to the addition of HCoV-229E at an MOI of 0.05. At 4 d.p.i., the resultant supernatants were subjected to the end point assay and TCID50 determination at 5 d.p.i.; the remaining cells (at 4 d.p.i.) were stained by crystal violet for visualization of cytopathic effects prior to being dissolved in 100% MeOH for quantification of the absorbance at 560 nm. The viabilities of MRC-5 cells (cultured in medium containing 10% FBS) and CC_50_ values were assayed and determined as for the MRC-5 cells (*vide supra*).

### End Point Dilution Assay for Determining Virus Titers

The end point dilution experiment was performed as previously described (Yang et al., 2017a). Viral titers of the supernatants obtained from the culture of HCoV-OC43 infected HCT-8 cells or HCoV-229E infected MRC-5 (MOI of 0.05) treated with the indicated compounds or vehicle DMSO were determined using an end point dilution assay. The Reed Muench method was used to determine TCID50 (Tissue Culture Infective Dose). The virus titer was then calculated as 1 TCID = 0.69 PFU (Plaque-Forming Units) (Reed and Muench, 1938).

### mRNA Isolation and RT-qPCR

These experiments were performed as described (Yang et al., 2007b). Mock or HCoV-229E infected (MOI of 1) lysates were processed and prepared at 30 h.p.i. without or with compound treatment. The total RNA was extracted with TRIzol reagent (Invitrogen). The relative viral RNA expression levels were determined by semi-RT-qPCR, analyzed with the ImageJ Analyzer program (http://imagej.nih.gov/ij/index.html), and normalized with the housekeeping gene GAPDH. The primers used to amplify the PCR products were 5′-GGC​GAG​GTG​GAA​TTT​GTT​TA-3′ and 5′-ACC​TTT​AAG​CCA​CCA​TGT​GC-3′ for ORF1; 5′-TCT​GCC​AAG​AGT​CTT​GCT​CG-3′ and 5′-AGC​ATA​GCA​GCT​GTT​GAC​GG-3′ for ORF N. The amplification of target cDNA was conducted under the following conditions: 25 cycles of 95°C for 30 s, 55°C for 20 s, and 72°C for 30 s for ORF N; 29 cycles of 95°C for 30 s, 55°C for 20 s, and 72°C for 30 s for ORF1, and 35 cycles of 95°C for 30 s, 55°C for 20 s, and 72°C for 30 s for GAPDH. The final PCR products were subjected to electrophoresis on 2% agarose gel containing ethidium bromide along with DNA markers.

### CPE and IFA for SARS-CoV-2

CPE was performed as described (Wu et al., 2004). For IFA, Vero E6 cells (BCRC number: 60476; derived from ATCC CRL-1586) were treated with each compound at the indicated concentration for 1 h at 37°C. The cells were adsorbed with SARS-CoV-2 (TCDC#4) (sequence available on the GISAID website) at an MOI = 0.01 for 1 h at 37°C. After virus adsorption, the cells were washed with PBS and fresh medium containing each compound at the indicated concentration was added. After 2 days, cells were fixed with 4% paraformaldehyde and permeabilized with 0.5% Triton X-100. The cells were stained with anti-SARS-CoV-2 N protein antibody, provided by Dr. An-Suei Yang of the Genomics Research Center, Academia Sinica, and goat anti-human IgG-Alexa Fluor 488 (A11013, Invitrogen) (in green). The nuclei were counter stained with DAPI (in blue) (D1306, Invitrogen). The N protein expression was measured using a high-content image analysis system (Molecular Devices). The cell viability was determined by Cell Counting Kit-8 (CCK-8) (Sigma-Aldrich, cat #96992). EC_50_ and CC_50_ values were calculated by Prism software.

### Plaque Assay for SARS-CoV-2

A plaque assay was performed in triplicate in 24-well tissue culture plates. The Vero E6 cells were seeded in DMEM with 10% FBS and antibiotics 1 day before infection. SARS-CoV-2 was added to the cell monolayer for 1 h at 37°C. Subsequently, viruses were removed and the cell monolayer was washed once with PBS before covering with media containing 1% methylcellulose (Sigma, cat #M0387) and the test compounds at the indicated concentrations. After 5–7 days, cells were fixed with 10% formaldehyde solution (Marcon™ Chemicals, cat #H121-08) overnight. After removal of overlay media, the cells were stained with crystal violet and the plaques were counted. The percentage of inhibition was calculated as [1 – (VD/VC)] × 100%, where VD and VC refer to the virus titer in the presence and absence of the test compound, respectively.

## Results

### The Tylophorine-Based Compounds and Cardenolides Tested Were Potent Inhibitors of FIPV and HCoV-OC43 Replication

**FIGURE 1:**
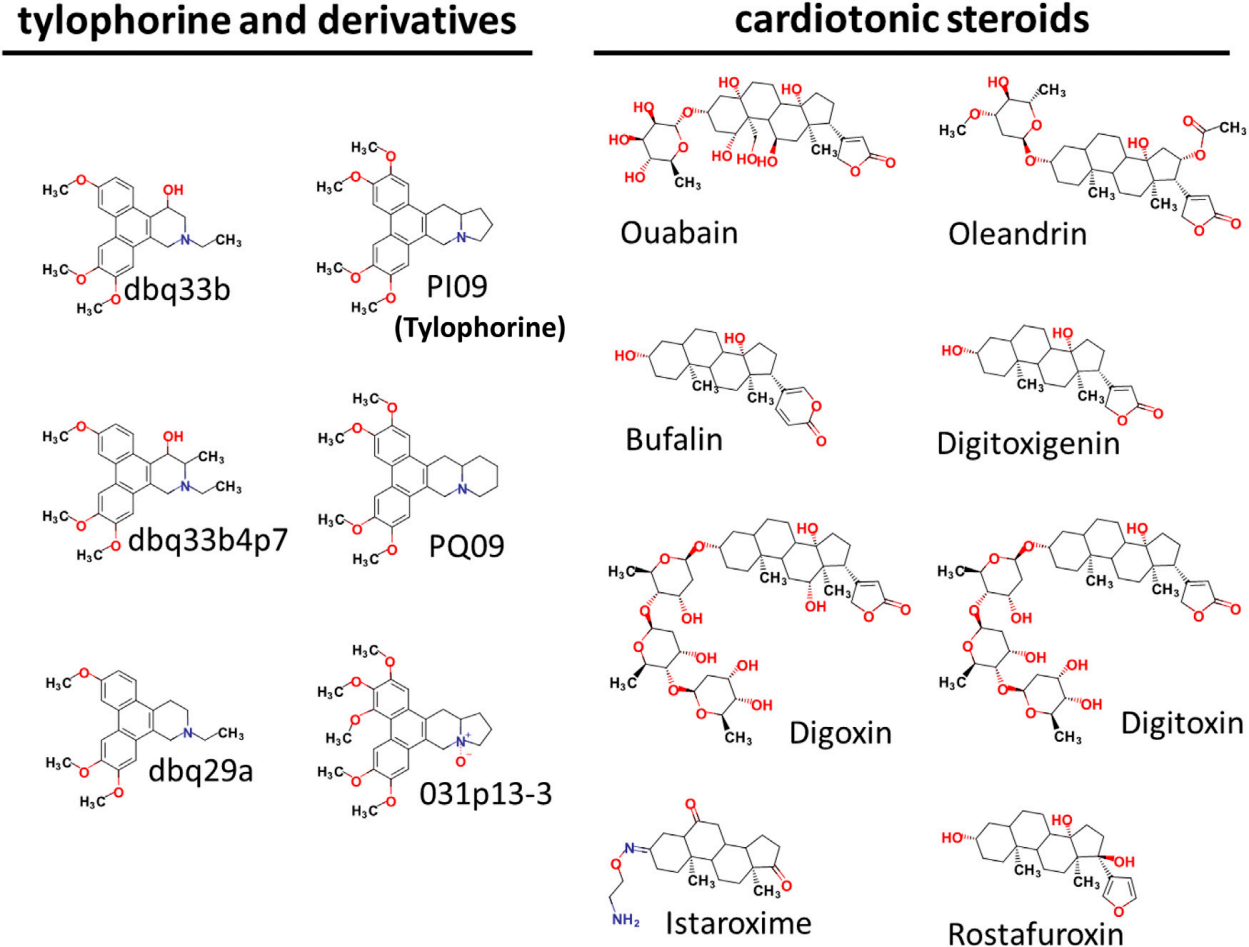
Chemical Structures of tylophorine-based compounds and cardiotonic steroids.

**FIGURE 2:**
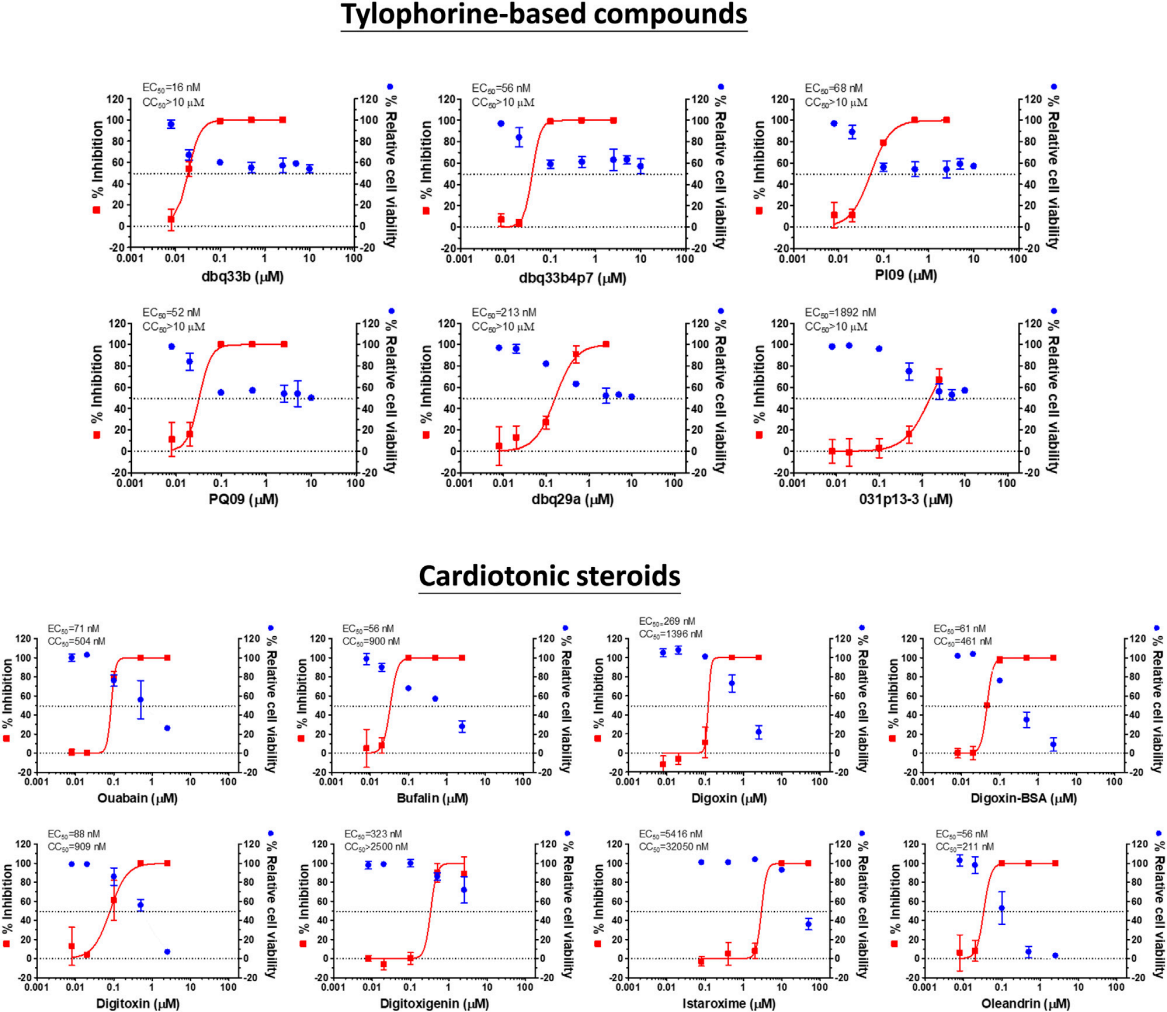
Dose response curves of tylophorine-based compounds and cardiotonic steroids on the viral inhibition in HCoV-OC43 infected HCT-8 and cell viability.

Tylophorine-based compounds have a plethora of biological activities including anti-inflammatory, anti-cancer, and anti-viral activities (Yang et al., Yang et al., 2006; Yang et al., 2007a; Wu et al., 2009; Yang et al., 2010; Lee et al., 2011; Lee et al., 2012; Yang et al., 2013; Qiu et al., 2015; Yang et al., 2017b; Lee et al., 2020). Cardenolides and bufadienolides are steroids incorporating a five- or six-membered lactone ring, and are best known for their inhibition of the Na^+^/K^+^ ATPase (Agrawal et al., 2012; Yang et al., 2017a), although they have a multitude of other biological activities as well, and may be useful for treating cardiac arrhythmias and human cancers, and reducing viral production (Prassas and Diamandis, 2008; Su et al., 2008; Diederich et al., 2017; Yang et al., 2017a). We identified six tylophorine-based compounds and eight cardiotonic steroids (seven cardenolides and one bufadienolide) (Figure 1. Chemical structures), and assessed their inhibition of FIPV (*alphacoronavirus*) by visual observation of cytopathic effects, and of HCoV-OC43 (*betacoronavirus*) by an immunofluorescent assay (IFA) against HCoV-OC43 nucleocapsid (N) protein. EC_50_ values for the tylophorine-based compounds ranged from 8 nM to 1.6 μM against FIPV and 16 nM to 1.9 μM against HCoV-OC43, with selectivity indices ranging from 610 to 5.3 (Table 1). EC_50_ values for the cardiotonic steroids ranged from 19 nM to 2.2 μM for FIPV and 56 nM to 5.4 μM for HCoV-OC43, with selectivity indices ranging from 28.5 to 3.3 (Table 1; Figure 2). In addition, the ouabain antagonist rostafuroxin did not exhibit any inhibitory activity against either FIPV or HCoV-OC43 at concentrations of up to 50 μM. Three tylophorine-based compounds (dbq33b, dbq33b4p7, and PI09) were highly potent inhibitors of HCoV-OC43, with EC_50_ values of 16 ± 4.7 nM, 56 ± 6.2 nM, and 68 ± 2.7 nM, respectively. In addition, two cardenolides (ouabain and oleandrin) and a bufadienolide (bufalin) were also potent inhibitors of HCoV-OC43, with EC_50_ values of 71 ± 7 nM, 56 ± 5 nM, and 56 ± 4 nM, respectively. All six of these compounds were then tested for their effect on HCoV-OC43 viral yields/titers.

**TABLE 1:** The inhibitory activities of tylophorine-based compounds and cardenolides against FIPV and HCoV-OC43.

Compound name	Feline infectious peritonitis virus^a^			Human coronavirus OC43^a^		
	EC_50_ (nM)^b^	CC_50_ (nM)^c^	Selectivity index	EC_50_ (nM)^b^	CC_50_ (nM)^c^	Selectivity index
	CPE/visualization	Visualization		(IFA)	(MTS)	
dbq33b	8 ± 2	3,125 ± 1,083	390.6	16 ± 5	>10,000	>610
dbq33b4p7	20 ± 7	6250 ± 2165	312.5	56 ± 6	>10,000	>179
PI09	62 ± 25	6375 ± 2250	102.8	68 ± 3	>10,000	>147
PQ09	17 ± 6	3094 ± 886	182.0	52 ± 6	>10,000	>193
dbq29a	352 ± 77	4313 ± 1125	12.3	213 ± 62	>10,000	>46.9
031p13-3	1,556 ± 638	>50,000	>32.1	1892 ± 228	>10,000	>5.3
Ouabain	1,556 ± 638	6250 ± 2165	4.0	71 ± 7	504 ± 11	7.1
Bufalin	19 ± 9	542 ± 213	28.5	56 ± 4	900 ± 180	16.1
Digoxin	212 ± 96	1188 ± 603	5.6	269 ± 45	1396 ± 350	5.2
Digoxin-BSA	115 ± 36	669 ± 242	5.8	61 ± 1	461 ± 129	10.0
Digitoxin	97 ± 6	825 ± 195	8.5	88 ± 16	909 ± 123	10.3
Digitoxigenin	458 ± 18	3563 ± 375	7.8	323 ± 20	>2500	>7.7
Istaroxime	2,156 ± 563	7125 ± 750	3.3	5416 ± 268	32,050 ± 1484	5.9
Oleandrin	729 ± 244	2847 ± 1069	3.9	56 ± 5	211 ± 62	3.8
Remdesivir^d^	ND^e^	ND	ND	947 ± 320	>50,000	>52.8
GS441524^d^	ND	ND	ND	6,929 ± 430	>50,000	>7.2

a Data are means ± SD from three rounds of experiments, each in triplicate (FIPV); and means ± SD from three independent experiments, each in duplicate (HCoV-OC43).

b EC50: The values of 50% maximal effective concentration.

c CC50: The values of 50% maximal cytotoxic concentration.

d Remdesivir used as a control reference compound.

e ND, not determined.

### Tylophorine-Based Compounds and Cardenolide Compounds All Reduced HCoV-OC43 Virus Titers in a Dose Dependent Manner but the Magnitude of Their Inhibition Was Highly Variable

**FIGURE 3:**
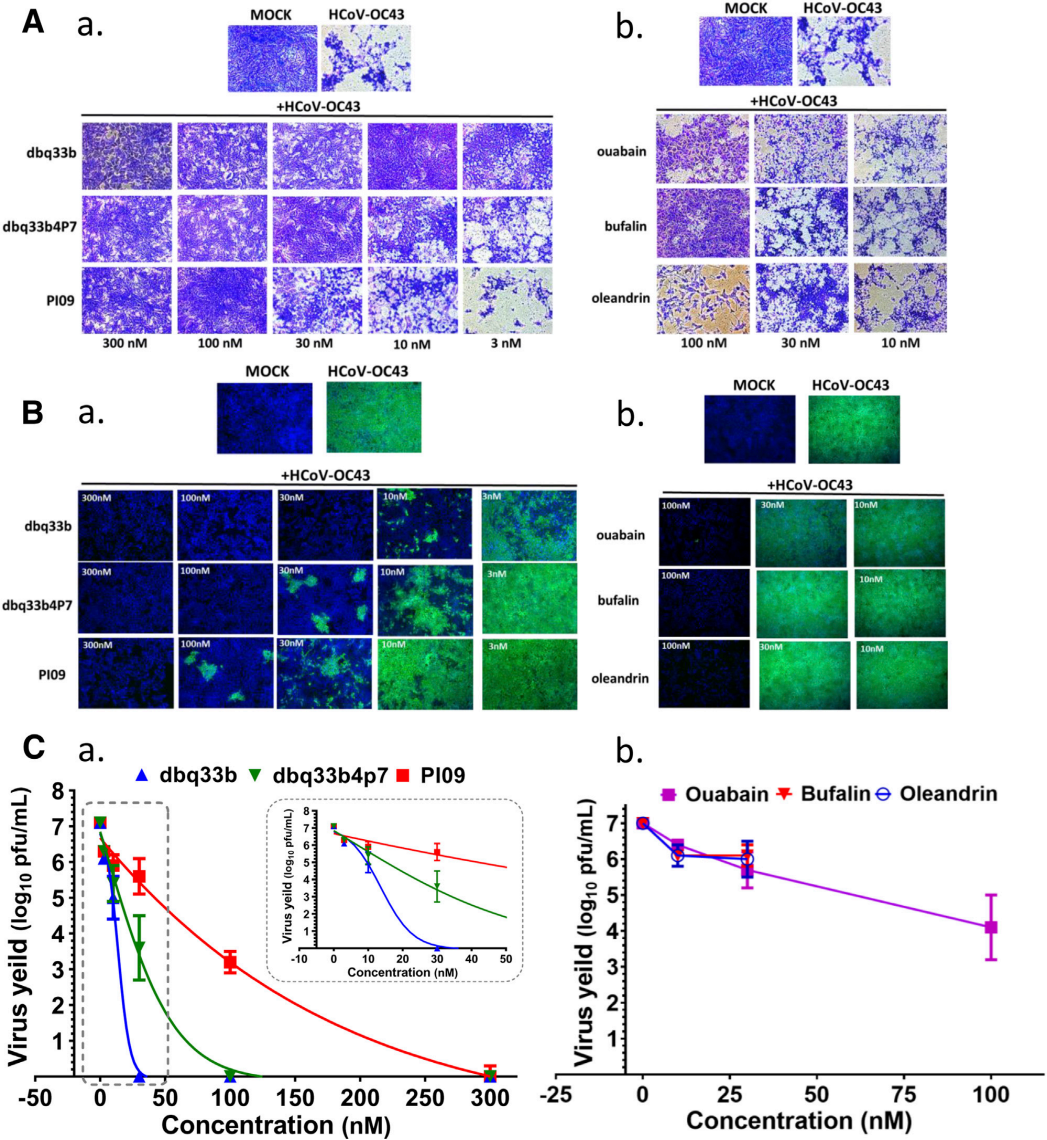
Anti-HCoV-OC43 activities of tylophorine-based compounds and cardenolides. **(A)** HCoV-OC43-induced cytopathy in HCT-8 cells protected by tylophorine-based compounds **(A-a)** or cardiotonic steroids **(A-b)**. Cells were stained by crystal violet for visualization of cytopathic effects. **(B)** The immunofluorescent assays of tylophorine-based compounds **(B-a)** and cardenolide compounds **(B-b)** against HCoV-OC43 N (green) and Hoechst staining (blue) for the host live cells. **(C)** Dose dependent effects of tylophorine-based compounds **(C-a)** and cardenolide compounds **(C-b)** on reducing HCoV-OC43 yields/titers. Indirect immunofluorescent assay (IFA) with antibodies against N protein (green) of HCoV-OC43 in HCoV-OC43 (0.05 MOI) infected HCT-8 cells at 72 h.p.i. treated with vehicle (0.5% DMSO) or chemicals as indicated. Nuclei (blue) were stained with Hoechst dye. HCT-8 cells were seeded the day before compound treatment or HCoV-OC43 infection. The tested compounds were added to the wells 1 h prior to the addition of HCoV-OC43 at an MOI of 0.05. The resultant cultures were then incubated for an additional 72 h at 37°C. The supernatant of cells in each specific treatment was collected and subjected to viral titer determination via an end-point dilution assay conducted with HCT-8 cells at 6 d.p.i. Images shown are representative of three independent experiments.

The inhibition of HCoV-OC43 by three representative tylophorine-based compounds (dbq33b, dbq33b4p7, and PI09) and representative cardiotonic steroids (ouabain, oleandrin, and bufalin) was assessed by visual observation of their cytopathic effects at 6 d.p.i. (Figure 3A) and assessed by IFA against HCoV-OC43 N protein at 3 d.p.i. (Figure 3B), with a subsequent end point assay and TCID50 determination, to establish if these inhibitory activities on viral titers were dose dependent.

End point assays were conducted to measure the titers remaining in supernatants of HCoV-OC43 infected HCT-8 cells treated with various concentrations of tylophorine-based compounds and cardiotonic steroids. After HCoV-OC43 infection at an MOI of 0.05 for 72 h, viral yields reached ∼10^7^ p.f.u./ml. The tylophorine-based compounds dbq33b, dbq33b4p7, and PI09 all significantly blocked viral replication and diminished viral yields; complete reduction of viral yield by ∼7–8 log of magnitudes was accomplished at concentrations of 30, 100, and 300 nM, respectively (Figure 3C-a). Ouabain, oleandrin, and bufalin also blocked viral replication and diminished viral yields, but only by a maximum of ∼2–3 orders of magnitudes [at concentrations of 10, 30, and 100 nM (Figure 3C-b), respectively].

### Tylophorine-Based Compounds Significantly Reduced HCoV-229E Virus Titers and Suppressed Viral Genome Replication in a Dose Dependent Manner

The activities of the tylophorine-based compounds (dbq33b, dbq33b4p7, and PI09) against another human coronavirus, HCoV-229E (an alpha-coronavirus), in fetus lung fibroblast MRC-5 cells were also ascertained.

**FIGURE 4:**
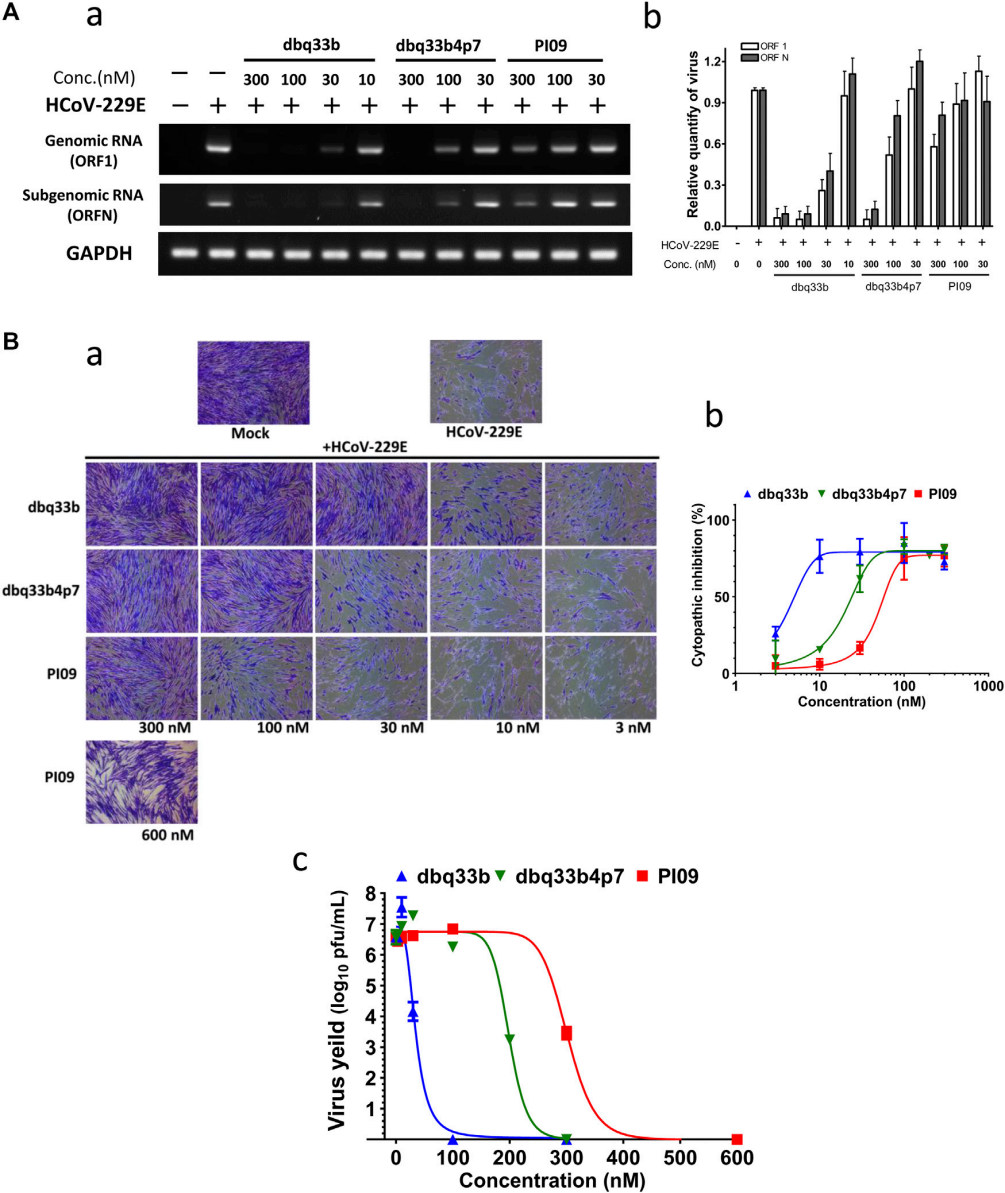
Anti-HCoV-229E activities of tylophorine-based compounds. **(A)** Tylophorine-based compounds significantly diminished the viral RNA levels in HCoV-229E infected MRC-5 cells in a dose dependent manner. Mock or HCoV-229E infected (MOI of 1) lysates were processed and prepared at 30 h.p.i. without or with compound treatment as indicated. The total RNA was extracted with TRIzol reagent (Invitrogen). The relative viral RNA expression levels were determined by semi-RT-qPCR, analyzed with the ImageJ Analyzer program (http://imagej.nih.gov/ij/index.html), and normalized with the housekeeping gene GAPDH. The final PCR products were subjected to electrophoresis on 2% agarose gel containing ethidium bromide along with DNA markers and the representative from three independent experiments was shown in the left panel **(A-a)**. Averages ± SD of relative expression levels from three independent experiments were shown in the right panel **(A-b)**. **(B)** Tylophorine-based compounds mitigated cytopathy and eliminated viral yields of HCoV-229E in infected MRC-5 cells. Cytopathic effects in MRC-5 cells induced by HCoV-229E were mitigated by tylophorine-based compounds **(B-a)** and the averages ± SD from three independent experiments were also shown **(B-b)**. The reduction of HCoV-229E yields/titers depended on the dose of the tylophorine-based compound that was present **(B-c)**. MRC-5 cells were seeded the day before compound treatment and HCoV-229E infection. The tested compounds were added to the wells 1 h prior to the addition of HCoV-229E at an MOI of 0.05. At 4 d.p.i. the resultant supernatants were subjected to the end point assay and TCID50 determination at 5 d.p.i.; the remaining cells were stained by crystal violet for visualization of cytopathic effect prior to being dissolved by 100% MeOH for quantitation of the absorbance at 560 nm. Shown are averages ± SD from three independent experiments.

First, HCoV-229E viral genome replication was examined by RT-PCR with specific primers against its open reading frame 1 (ORF1) and ORF nucleocapsid (ORFN). MRC-5 cells were inoculated with HCoV-229E at an MOI of 1, and the infected cells with or without drug treatment were analyzed at 30 h.p.i. by RT-PCR with specific primers. Results shown that dbq33b, dbq33b4p7, and PI09 inhibited HCoV-229E viral genome replication and subgenomic viral RNA syntheses at concentrations of 10–300 nM in a dose dependent manner (Figure 4A).

Second, the cytopathic effects of HCoV-229E on infected MRC-5 cells (MOI of 0.05) at 4 d.p.i. were also visualized (Figure 4B-a) and the resultant supernatants of each compound treatment were subjected to the TCID50 end point assays. Cells were stained with crystal violet, to determine the EC_50_. In addition, CC_50_ values were also determined, and selectivity indices calculated (Figure 4B-b; Table 2).

**TABLE 2:** The inhibitory activities of tylophorine-based compounds against HCoV-229E and SARS-CoV-2.

Compound name	Human coronavirus 229E^a^			SARS-CoV-2^a^					
	EC_50_ (nM)^b^	CC_50_ (nM)^c^	Selectivity index	EC_50_ (nM)^b^	CC_50_ (nM)^c^	Selectivity index	EC_50_ (nM)^b^	CC_50_ (nM)^c^	Selectivity index
	CPE^d^/crystal violet	MTS		CPE^d^/visualization	Visualization		IFA^e^	CCK-8	
dbq33b	6.5 ± 1.0	1,712 ± 161	264	2.5	1,250	500	13.9	5,104	367
dbq33b4p7	25.4 ± 3.1	7,362 ± 288	290	20	2,500	125	31.9	2,973	93
PI09	71.4 ± 13.2	6,635 ± 578	93	78	5,000	77	76.8	3,546	46

a Data are means mean ± SD from three independent experiments, each in duplicate (HCoV-229E); and means from duplicate for SARS-CoV-2.

b EC50: The values of 50% maximal effective concentration which were determined by CPE by crystal staining measurement or by visualization and by IFA as indicated.

c CC50: The values of 50% maximal cytotoxic concentration which were determined by MTS, visualization or cell counting CCK-8 as indicated.

d CPE: cytopathic effect by visualization (for SARS-CoV-2) or crystal violet stained and correspondent absorption measured at 560 nm (HCoV-229E).

e IFA: immunoflurorescent assay with antibody against virus N-protein.

Third, it was found that HCoV-229E viral yields reached 10^6^–10^7^ p.f.u./ml at 5 d.p.i, and treatments with dbq33b, dbq33b4p7, or PI09 all significantly blocked viral replication and eliminated viral yields, resulting in a complete reduction of viral yield by 6–7 log of magnitudes at concentrations of 100 nM, 300 and 600 nM (Figure 4B-c), respectively in HCoV-229E infected MRC-5 cells.

### Tylophorine-Based Compounds Show Potent anti-SARS-CoV-2 Activities in Vero E6 Cells

**FIGURE 5:**
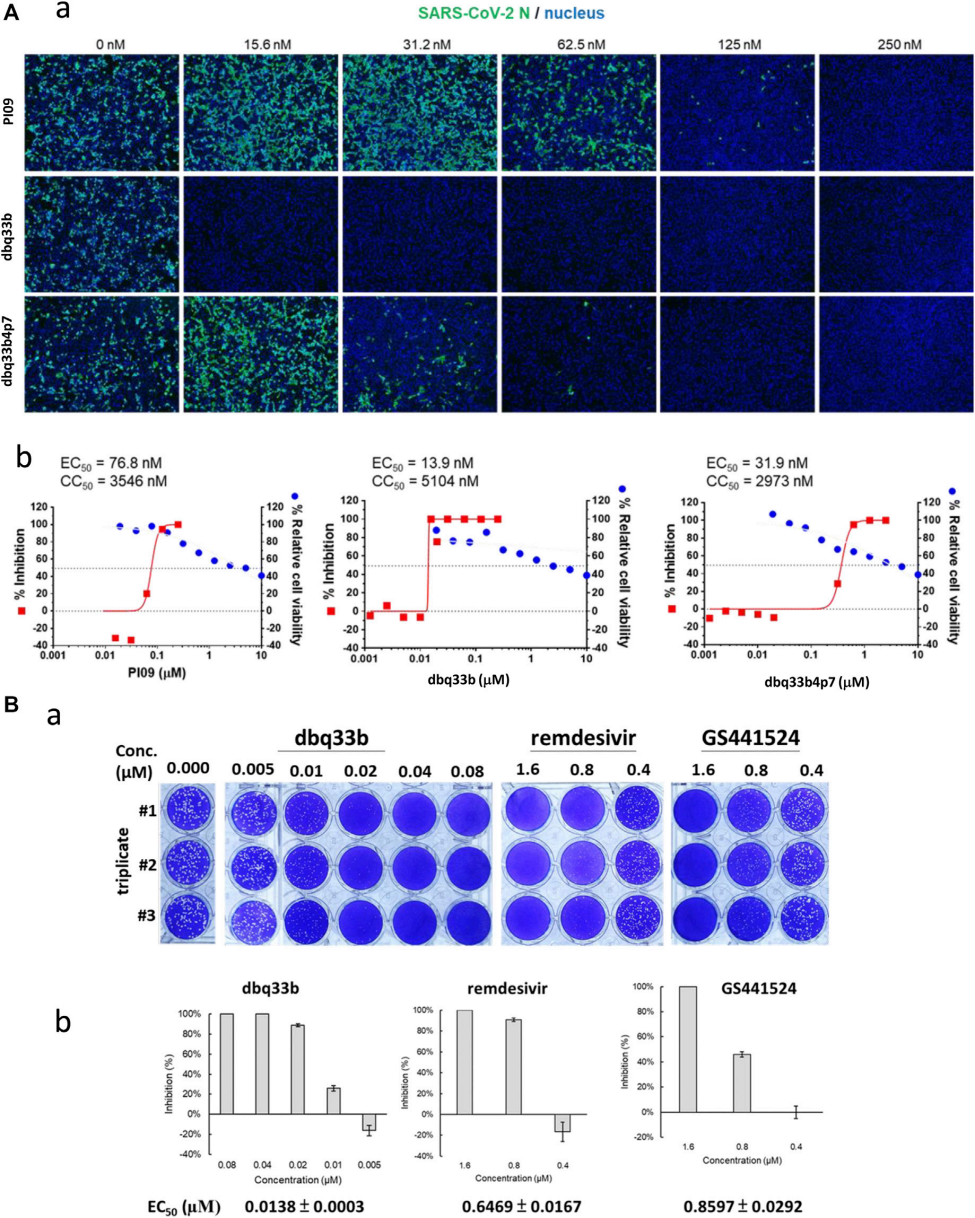
Anti-SARS-CoV-2 activities of tylophorine-based compounds. **(A)** The immunofluorescent assays against SARS-CoV-2 N (green) and DAPI staining (blue) for the Vero E6 host live cells. Vero E6 cells were treated with each compound at the indicated concentrations for 1 h at 37°C. The cells were adsorbed with SARS-CoV-2 (TCDC#4) at MOI = 0.01 for 1 h at 37°C. After virus adsorption, the cells were washed with PBS and fresh medium with each compound added at the indicated concentrations and then incubated for 2 days. The cells were fixed with 4% paraformaldehyde and permeabilized with 0.5% Triton X-100. The cells were stained with anti-SARS-CoV-2 N protein antibody and anti-human IgG-488 (green) and the nuclei were counter stained with DAPI (blue) as shown **(A-a)**. The N protein expression was measured by using a high-content image analysis system (Molecular Devices); the cell viability was determined by Cell Counting Kit-8 (CCK-8); and EC_50_ and CC_50_ were calculated by Prism software **(A-b)**. Shown results are representative of two independent experiments. **(B)** Tylophorine-based compound dbq33b profoundly diminished the plaque formation caused by SARS-CoV-2 in Vero E6 cultured plaque assays. Plaque assay was performed in triplicate in 24-well tissue culture plates. The Vero E6 cells were seeded in DMEM with 10% FBS and antibiotics 1 day before infection. SARS-CoV-2 was added to the cell monolayer for 1 h at 37°C. Subsequently, viruses were removed and the cell monolayer was washed once with PBS before covering with media containing 1% methylcellulose (Sigma, cat #M0387) and test compounds at indicated concentrations for 5–7 days. The cells were fixed with 10% formaldehyde solution (Marcon™ Chemicals, cat #H121-08) overnight. After removal of overlay media, the cells were stained with crystal violet and the plaques were counted **(B-a)**. Remdesivir and GS441524 were used as control reference compounds. The percentage of inhibition was calculated as [1 – (VD/VC)] × 100%, where VD and VC refer to the virus titer in the presence and absence of the test compound, respectively **(B-b)**. Shown results are representative of two independent experiments.

The tylophorine-based compounds dbq33b, dbq33b4p7, and PI09 were examined for their inhibitory activity against SARS-CoV-2 in Vero E6 cells. The cytopathic effects of SARS-CoV-2 on Vero E6 cells in the presence of the test compounds at a two-fold dilution and a series of 12 concentrations were visualized, and values of EC_50_ and CC_50_ calculated (Table 2). All three compounds were found to be highly potent inhibitors of SARS-CoV-2, with EC_50_ values of 2.5, 20, and 78 nM, respectively (Table 2). In addition, an IFA assay at 2 d.p.i. was also performed using an antibody against SARS-CoV-2 N protein; the corresponding EC_50_ values were determined to be 14, 32, and 77 nM respectively – comparable to those obtained by cytopathy assay. As expected, dbq33b, dbq33b4p7, and PI09 were highly potent with selectivity indices of 376, 93, and 46, respectively (Table 2; Figure 5A).

The inhibitory potency of dbq33b, the most potent compound, was additionally determined by plaque assay in SARS-CoV-2 infected Vero E6 cells. An EC_50_ of 14 nM was obtained (Figure 5B), consistent with the cytopathic effect assay and IFA results (Table 2).

## Discussion

Coronaviruses are grouped into four genera: *Alphacoronaviruses*, *Betacoronaviruses*, *Gammacoronaviruses* and *Deltacoronaviruses*, of which those belonging to the former two are infectious to humans (Chen et al., 2020). The tylophorine-based compounds tested herein exerted potent, broad-spectrum antiviral activity against multiple types of coronavirus (Yang et al., 2010; Lee et al., 2012; Yang et al., 2017b), Tables 1 and 2; Figures 1–5), including SARS-CoV, SARS-CoV-2, HCoV-OC43, and MHV (*betacoronavirus*) and TGEV, FIPV, and HCoV-229E (*alphacoronavirus*). Accordingly, they should amongst the first compounds to be tested as therapies against novel coronaviruses in the future.

Basically, the structure-activities relationships of tylophorine-based derivatives and cardiotonic steroids have been proposed or discussed as published previously (Yang, et al., 2010; Lee, et al., 2012; Yang, et al., 2017a). The results obtained herein are in consistent with previous structure-activities relationships or conclusion.

The cardiac steroids tested were also highly potent inhibitors of these coronaviruses, but their safety indices were only up to ∼16 (for HCoV-OC43, assayed by IFA) and or ∼28 (for FIPV, assayed by visualization of CPE) (Table 1). Furthermore, the inhibition of HCoV-OC43 was limited to two to three magnitudes of viral load, as the p.f.u./ml decreased from ∼10^7^ to ∼10^5^ or 10^4^ (Figure 3C-b) by end point assay and TCID50 determination. On the contrary, the tylophorine based compounds were all highly potent inhibitors of all the coronaviruses tested, demonstrating complete elimination of viral load/yield by ∼10^7^ p.f.u./ml (Figure 3C-a) and good safety indices of up to >610 (for HCoV-OC43, assayed by IFA) and ∼390 (for FIPV, by visualization of CPE) (Table 1). Therefore, the tylophorine-based compounds tested are concluded to be inherently superior to the cardiac steroids for the purpose of combating COVID-19. These differences may reflect their differential underlying mechanisms and pharmacological targets – the tylophorine-based compounds targeting viral RNA (Yang et al., 2017b), and the cardiotonic steroids affecting host cell factors (Yang et al., 2017a; Yang et al., 2018).

The tylophorine-based compounds also had the property of cell growth inhibition, with the selectivity index depending on the design of the experiment and the methods used to measure cytotoxicity (Yang et al., 2017a). Thus, selectivity indices of tylophorine compounds, CC_50_/EC_50_, may be easily unintentionally underestimated. Moreover, coronavirus-infected cells treated with tylophorine compounds usually expand their size (area in the culture plates) and are protected from the formation of syncytia or multinucleated giant cells resulting from virus-induced fusion of cell membranes (Yang et al., 2010; Lee et al., 2012).

Tylophorine-based compounds block viral replication as demonstrated in three ways: 1) by directly targeting and interacting with genomic/subgenomic RNAs and the nucleocapsid protein; 2) by colocalizing with coronaviral RNA and RNA-dependent RNA polymerase in the viral replication-transcription complexes (RTCs) surrounding nuclei of infected cells; and 3) by effectively blocking syntheses of coronaviral antigens and genomic/subgenomic RNAs (Yang et al., 2017b). Coronavirus N protein forms complexes with genomic and subgenomic RNAs and plays a crucial role in virus replication, transcription and translation (McBride et al., 2014). Furthermore, N protein exclusively binds to nonstructural protein 3 (NSP3), a component of RTCs, for recruitment to RTCs is crucial and critical for viral RNA syntheses (Cong et al., 2020). Therefore, by directly targeting viral replication-transcription machinery, tylophorine-based compounds are highly effective inhibitors of coronaviral replication. Accordingly, they merit further development into an approved therapeutic agent.

## Data Availability Statement

The raw data supporting the conclusions of this article will be made available by the authors, without undue reservation.

## Author Contributions

C-WY, Y-ZL, and H-YH performed most of the biochemistry, and molecular biology experiments. J-TJ, T-TP, J-JL, C-CL, T-LC, Y-HP, H-CK, C-PC, G-HN, and S-HW performed part of the biochemistry, and molecular biology experiments. S-JL, C-WY, Y-ZL, H-YH, J-TJ, Y-LL, and S-YC designed experiments and analyzed the obtained results; interpreted the data and wrote the manuscript. R-BY, W-ZH, J-HL, H-KS and C-TC were involved in composition of the manuscript. S-JL supervised the experimental design, the interpretation of the data, and the composition of the manuscript.

## Funding

This work was funded by the National Health Research Institutes, Taiwan and the Ministry of Science and Technology, Taiwan (grants of MOST 106-2320-B-400-009-MY3 and MOST 108-2811-B-400-511) as well as an emergent grant for COVID-19 from the Ministry of Health and Welfare, Taiwan.

## Conflict of Interest

The authors declare that the research was conducted in the absence of any commercial or financial relationships that could be construed as a potential conflict of interest.
